# Publisher Correction: Broad modulus range nanomechanical mapping by magnetic-drive soft probes

**DOI:** 10.1038/s41467-017-02555-4

**Published:** 2018-01-16

**Authors:** Xianghe Meng, Hao Zhang, Jianmin Song, Xinjian Fan, Lining Sun, Hui Xie

**Affiliations:** State Key Laboratory of Robotics and Systems, Harbin Institute of Technology, 2 Yikuang, Harbin, 150080 China

Correction to: *Nature Communications* 10.1038/s41467-017-02032-y, published online 05 December 2017

In the original version of this Article, the text labels on the bottom-right panel in Fig. 1c were inadvertently displaced during the production process. The correct version is:

 **Fig. 1**

which replaces the previous incorrect version :

 **Fig. 2**

This has now been corrected in both the PDF and HTML versions of the Article.

