# Supplementary material for: Broad modulus range nanomechanical mapping by magnetic-drive soft probes
Source: Nat Commun. 2017 Dec 5;8:1944. doi: 10.1038/s41467-017-02032-y (PMC5717272; doi:10.1038/s41467-017-02032-y)
Supplement: Supplementary file 1 — Supplementary Information [file 41467_2017_2032_MOESM1_ESM.pdf]

Supplementary Note 1. Brief Review of Magnetic Forces in Atomic Force Microscopy

The invention of Atomic Force Microscope (AFM) by Binnig and Quate in 1986 relied on the interaction forces between a sharp tip and the sample[1]. These atomic interaction forces are often expressed as long and short ranged attractive and repulsive forces which can be oversimplified as:

$$F_{\text{total}} = F_{\text{vdW}} + F_{\text{magnetic}} + F_{\text{electrostatic}} + F_{\text{chemical}} \quad (1)$$

Each of these major components of the total interaction force, dominates certain disciplines either individually or in various combinations with others. Among all, magnetic forces have attracted arguably the most attention due to its significance to the basic sciences and industries like data storage. Thus, it did not take long for the development of first Magnetic Force Microscope (MFM) in 1987[2]. After MFM's introduction, it was realized that the magnetic forces can be used to bend the cantilever directly. This idea was merged with dynamic AFM modes which oscillates the cantilever above the sample surface for non-contact or intermittent contact operations. Unlike MFM which uses a magnetic tip which bends the cantilever due to its interaction with the sample, direct cantilever excitation with magnetic forces employs a solenoid which generates an external magnetic field[3]. Cantilever excitation can be achieved with the force generated by the external magnetic field gradient or with the torque of a perpendicular magnetic field. Many researchers worked with magnetic cantilevers either by coating them with magnetic materials such as cobalt via thin film deposition methods or gluing small magnets to the backside of the cantilever[4-6]. Their motivation was to achieve a better signal-to-noise ratio thus increase the resolution which has been demonstrated with other direct probe actuation methods[7,8] compared to indirect (where all the cantilever holder setup is oscillated) actuation methods. With indirect actuation methods the drive spectrum forms a forest of peaks due to multiple resonances of all the components shaken in the cantilever holder setup to oscillate the cantilever[9]. This way the cantilever's resonance peak can easily be dominated, merged or mistaken with other peaks.

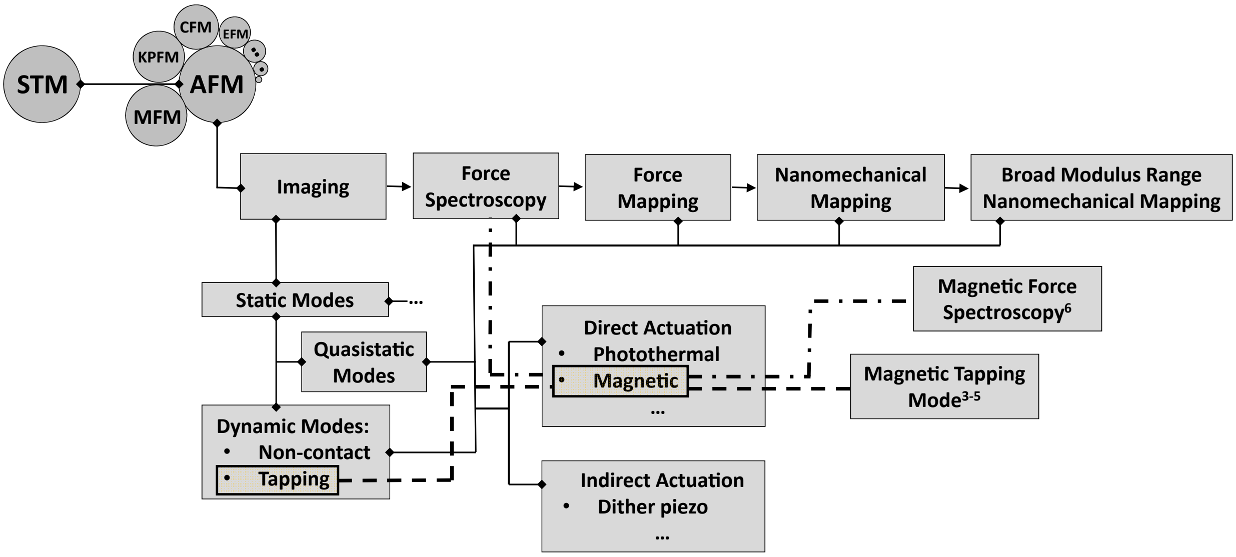

Supplementary Figure 1. Corner stones of AFM development and the use of magnetic forces.

Whereas with direct excitation provides a single clean peak. Considering that the actual total force sensed by the cantilever is most certainly not only the above mentioned forces but includes much more and often divided into three main components i.e.: the interaction, the hydrodynamic, and the inertial[10]. Here, the interaction accounts for the above mentioned forces as well as friction forces, tip wear etc. Thus, in reality the nature of the total force measured by AFM is quite complex. A lot of effort has been invested in trying to reduce and remove any irrelevant contribution to the forces of interest. This is where direct excitation methods get the spot light. Supplementary Fig. 1 summarizes the corner stones of AFM development, AFM operation modes based on cantilever dynamics and the use of magnetic forces. Each corner stones can contain multiple sub-categories, however, due to relevance and spatial limitations they are not shown.

Supplementary Note 2. Derivation of the relationship between two deflection sensitivities

The force F and torque M apply to the cantilever free end (Supplementary Fig. 2), which represents the tip position, respectively, so that the deflection curves of the cantilever are as follows:

$$z(x,t) = \frac{Fx^2}{6EI}(3L - x) \tag{2}$$

$$z(x,t) = \frac{Mx^2}{2EI} \tag{3}$$

where E is the elastic modulus, I is the moment of inertia with respect to x-axis, L is the length of the cantilever. The slope of each point on these two deflection curves can be described as

$$\frac{\partial z(x,t)}{\partial x} = \frac{Fx(2L - x)}{2EI} \tag{4}$$

$$\frac{\partial z(x,t)}{\partial x} = \frac{Mx}{EI} \tag{5}$$

The photodiode signals U_F,U_M are proportional to the displacement at the free end L and the slope of the cantilever at the laser spot position L_{laser}. Thus, the photodiode signal can be given by

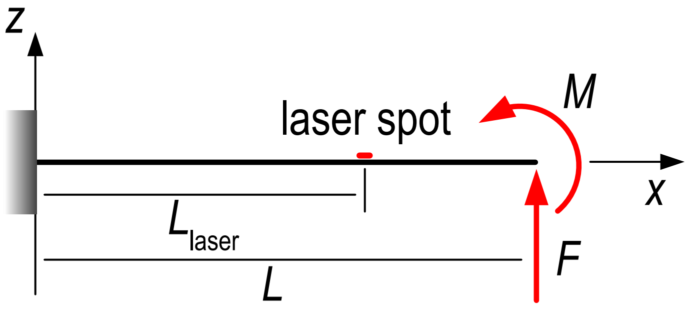

Supplementary Figure 2. Scheme of the cantilever applied by force and torque on the free end.

$$U_F = \gamma_F \, z(x,t)|_{x=L} = \beta_{\text{slope}} \left. \frac{\partial z(x,t)}{\partial x} \right|_{x=L_{\text{laser}}} \tag{6}$$

$$U_M = \gamma_M \, z(x,t)|_{x=L} = \beta_{\text{slope}} \left. \frac{\partial z(x,t)}{\partial x} \right|_{x=L_{\text{laser}}} \tag{7}$$

where γ_F is the force deflection sensitivity, γ_M is the torque deflection sensitivity, β_{slope} is the angular sensitivity. Combing those equations mentioned above, the relationships between two deflection sensitivities and angular sensitivity are

$$\gamma_F = \frac{3L_{\text{laser}}(2L - L_{\text{laser}})}{2L^3} \beta_{\text{slope}} \tag{8}$$

$$\gamma_M = \frac{2L_{\text{laser}}}{L^2} \beta_{\text{slope}} \tag{9}$$

From supplementary eqs 8 and 9, we see that β_{slope} establishes a bridge for solving the relationship between γ_F and γ_M. Therefore, that relationship can be expressed as

$$\gamma_M = \frac{4}{3(2 - \widetilde{L})} \gamma_F \tag{10}$$

Supplementary eq 10 expresses the transformation of the force deflection sensitivity into the torque deflection sensitivity in terms of the length ratio $\widetilde{L} = L_{\text{laser}}/L$. When $\widetilde{L} = 2/3$, those two sensitivities are equal. This means that we can obtain the displacement of the probe tip directly from the measured signal.

Supplementary Note 3. Magnetic Drive Strength

In Figure 2a of the main context, a force modulation probe (B-lever of HQ:NSC36/No Al, nominal spring constant of $k = 2$ N m^{-1}) is used to estimate the equivalent magnetic driving force A_{f} that is calculated by:

$$A_{\text{f}} = kA_{\text{m}} \sqrt{\left[1 - \left(\frac{\omega}{\omega_o}\right)^2\right]^2 + \left(\frac{1}{Q}\frac{\omega}{\omega_o}\right)^2} \tag{11}$$

where A_{m}, ω_o and Q are the the off-resonance (ω) oscillation amplitude, the first resonant frequency and quality factor of the probe, respectively. For the measurement in air, this calculation is simplified as $A_{\text{f}} = kA_{\text{m}}$ when $\omega \ll \omega_o$ and $Q \gg 1$ ($Q = 210$ for the testing probe in air).

The magnitude of the magnetic driving force is proportional to the cube of the diameter of the magnetic bead. Larger magnetic force is generated by the magnetic bead with a size of Ø11.4 μm, while a magnetic bead with a size of Ø3.8 μm is used to drive the 0.006 N m^{-1} probe in liquid. Considering the liquid damping ($Q \approx 1$ for this probe) and the inertial effects, the magnetic driving force at 250 Hz is calibrated from the above equation, as shown in Supplementary Fig. 3. A peak-to-peak current of ~ 6.9 mA is applied to the solenoid to oscillate the probe with an amplitude of 260 nm (equivalent force: 1.54 nN).

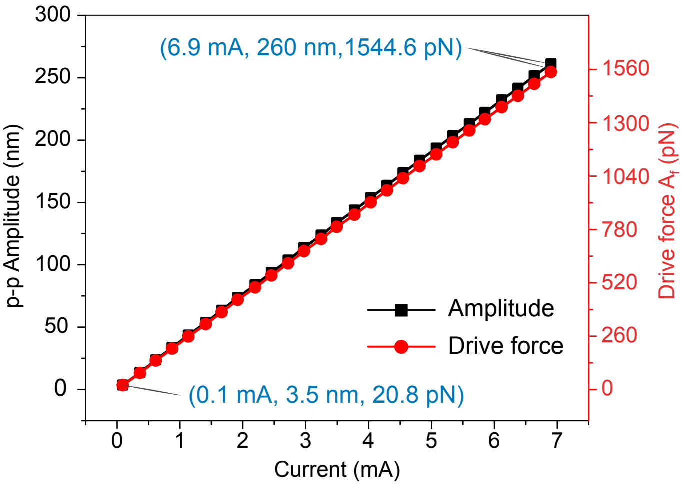

Supplementary Figure 3. Magnetic drive strength of Probe II. Calibrated magnetic drive force (A_f) when the probe (0.006 N m^{-1} with a Ø3.8 μm magnetic bead) is oscillated at 250 Hz with different driving currents (A_c) in water.

Supplementary Note 4. NM of Bacteria in Liquid

NM of Finegoldia Magna bacteria (immobilized on the glass slide) is performed in deionized water (25 ^{o}C) using Probe I which is driven at 1 kHz and with the amplitude of 100 nm. The probe dynamics calculations took into account of the largely reduced first resonant frequency (29.5 kHz) of the probe in water. Prior to scanning, a single force-distance curve test was performed to determine the peak force setpoint of 8 nN. The magnetic drive force was recorded as the probe tip became very close to the sample surface to remove the hysteresis effect caused by hydrodynamic damping in the liquid media.

The topography image of the Finegoldia Magna bacteria is shown in Supplementary Fig. 4a, and the corresponding cross section in Supplementary Fig. 4e shows the bacterium has a height of 600–650 nm. The adhesion (Supplementary Fig. 4b) is quite low in liquid and ranged from 2–5.4 nN (Supplementary Fig. 4f), whereas the high-contrast maps of indentation and reduced elastic modulus showed the soft bacteria has a clear difference from the hard glass substrate with values centered at $\sim$ 5 nm and $\sim$ 230 MPa (Supplementary Fig. 4g and h), respectively. The background noise of the indention depth is greatly compressed to sub-nanometer which mainly due to the system noises, as well as softened or even floating coverings (proteins, lipids, DNA and other matter) on the glass substrate. Although the silicon probe is not capable of measuring the glass surface, as it is common sense, the clear and high-contrast elastic modulus map verifies the BMR NM capability.

Supplementary Note 5. Calculation of the Lateral Resolution

Apart from the *E. coli* TOP 10, another type of bacterium *E. coli* TSK (dried for more than 48 hours) has been tested with our method. As shown in Supplementary Fig. 5a, the bacterium cell has several flagella with a diameter varying from 4 to 10 nm (height profile in Supplementary Fig. 5d). Their structures

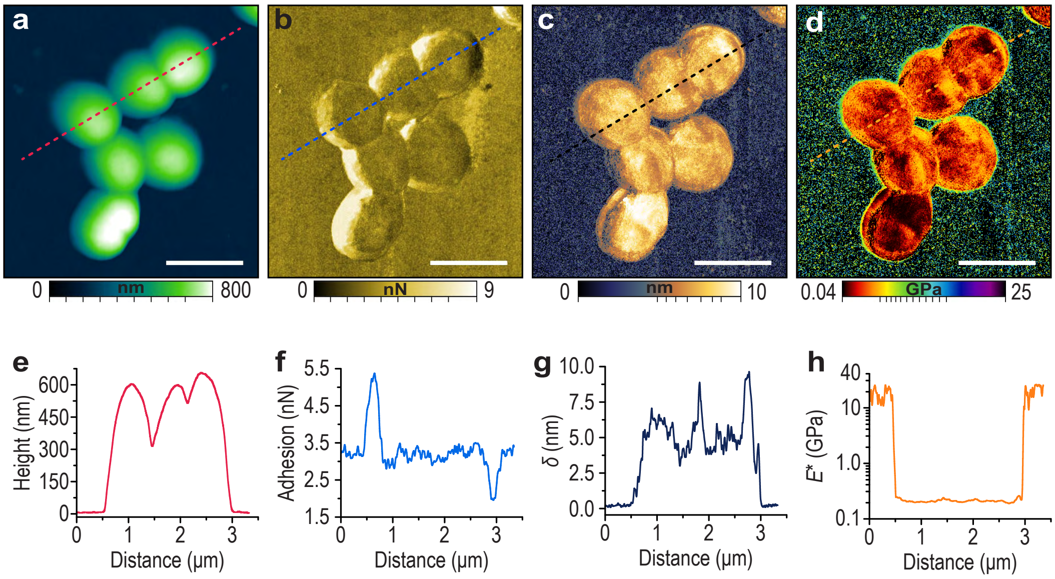

Supplementary Figure 4. Nanomechanical mapping of Finegoldia Magna bacteria in liquid. (**a**) Topography. Maps of (**b**) the adhesion force, (**c**) the indentation depth and (**d**) the reduced elastic modulus. (**e–h**) Corresponding cross-section profiles obtained from **a–d**, respectively. Scale bar, 1 μm.

can be clearly seen in the adhesion force map in Supplementary Fig. 5b with a difference of 13 nN. The elastic modulus map (Supplementary Fig. 5c) demonstrate that the bacteria flagella are clearly recognized from the silicon substrate that is covered with proteins, lipids, salt and other matter, and the cross section profile (bottom of Supplementary Fig. 5d) also proves a similar lateral resolution on the sample with discontinuous structures. The reduced elastic moduli of flagella is ranging from about 500 MPa to 1.5 GPa which is strongly affected by the substrate due to their small diameters.

To quantify the lateral resolution, as shown in Supplementary Fig. 6a (The grayscale range is properly adjusted to the height of flagella for clear display, which has no influence on the measurement results), statistical calculation is performed for lateral resolution with 40 measurements on different positions that are evenly distributed on flagella of the *E. coli* TOP10 cell (Figure 7 in the main context), and the average width of flagella to be 29.37±14.73 nm with max and min to be 76.7 and 13.8 nm, respectively. Similarly, as seen in Supplementary Fig. 6b (*E. coli* TSK cell), the average width of flagella (from 20 measurements) to be 24.88±4.5 nm with max and min to be 47.7 and 20.8 nm, respectively. These results, as can be seen in Figure 7 reveal that the BMR NM can be successfully applied with high resolution on a blend of soft and hard, continuous and discontinuous, linear and non-linear surfaces.

Supplementary Note 6. Calibration of the laser spot position

The key point of using supplementary eq10 is to calibrate the length ratio. From supplementary eqs 5 and 7, we can obtain when the laser spots are at L_{laser} and $L_{\text{laser}} + \Delta L$, where the photodiode signals U_M and

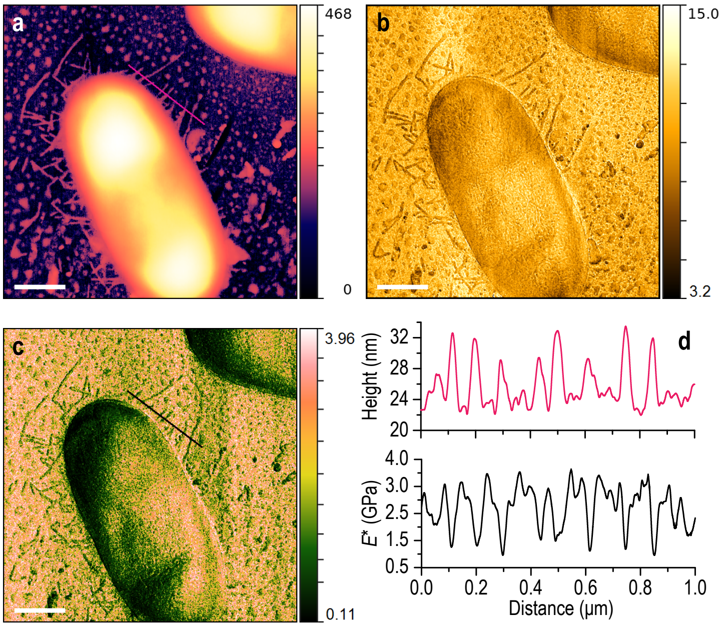

Supplementary Figure 5. High spatial resolution nanomechanical mapping of an *E. coli* TSK cell. (**a**) Topography (nm). Maps of (**b**) the adhesion force (nN) and (**c**) the reduced elastic modulus (GPa). (**d**) Profiles of the height (top) and reduced elastic modulus (bottom) obtained from **a** and **c**, respectively. Scale bar, 500 nm.

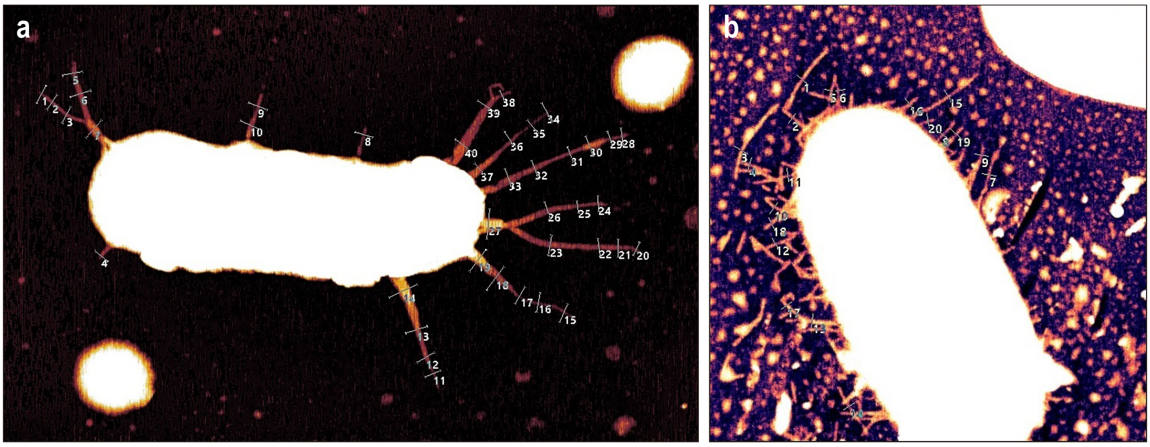

Supplementary Figure 6. Statistical calculation of the lateral resolution. (**a**) Statistical calculation of the lateral resolution with 40 different measurements on flagella of *E.coli* TOP10 cell. (**b**) Statistical calculation of the lateral resolution with 20 different measurements on flagella of *E.coli* TSK cell.

$U_M + \Delta U$ are described as

$$U_M = \beta_{\text{slope}} \frac{ML_{\text{laser}}}{EI} = \alpha L_{\text{laser}} \tag{12}$$

$$U_M + \Delta U = \beta_{\text{slope}} \frac{M(L_{\text{laser}} + \Delta L)}{EI} = \alpha (L_{\text{laser}} + \Delta L) \tag{13}$$

where α is a constant. Combing supplementary eqs 12 and 13, α can be described as

$$\alpha = \frac{\beta_{\text{slope}} M}{EI} = \frac{\Delta U}{\Delta L} \tag{14}$$

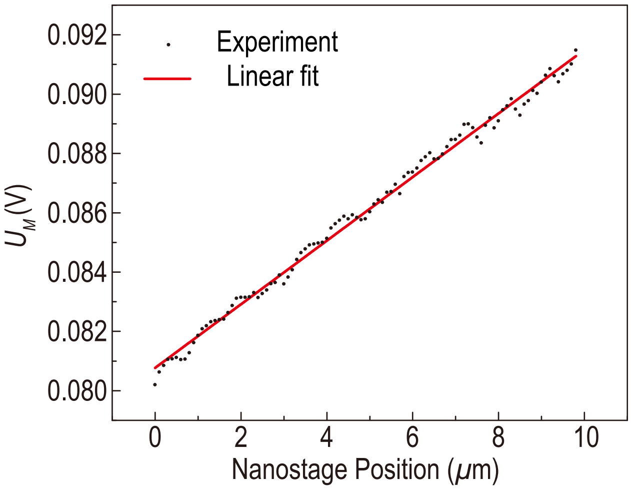

Supplementary Figure 7. Linear relationship between nanostage position and photodiode signal.

Therefore, the calibration process is as follows:

(1) Manually adjust the laser spot roughly at two-thirds of the probe length.

(2) Drive the coil to generate sinusoidal alternating magnetic field of 10Hz.

(3) Move the cantilever by a nanopositioning stage in the x-axis direction of 0.1μm step and record the amplitude of the voltage from photodiode.

(4) Record the position of nanostage X-axis (μm) and the photodiode signal $U_M(\text{V})$.

We can obtain the U_M-X scatterplot (Supplementary Fig. 7) and the slope of the linear fitting curve is the constant α. After obtaining the constant α, the laser spot position L_{laser} can be calculated by supplementary eq12. Therefore, the position of the laser spot can be precisely controlled to ensure that $\widetilde{L} = 2/3$.

Supplementary Note 7. Cantilever Preparation

Requirements of magnetic microbead probe preparation are similar to colloidal probe methods. These are: i) Glue deposition on the cantilever, ii) Placement of the bead, iii) Calibration of the spring constant after the glue is dried. In order to achieve these steps with haste, a pneumatic micromanipulation system is developed as shown in Supplementary Fig. 8a. The micromanipulation system mainly consists of a micro vacuum pump, a solenoid valve, a vacuum regulator and micropipettes (supported on a motorized stage with a motion resolution of 50 nm). Thanks to the top-view and side-view optical microscopes, glue (DP760 epoxy adhesive) and the microbead can be sequentially deposited and released on the target position with high precision, respectively. Supplementary Fig. 8b shows micropipettes with different aperture diameters which are used to manipulate magnetic microbeads with a diameter range of 3–15

μm. Supplementary Fig. 8c shows the optical microscopy images captured during the adhesive bonding process. One micropipette is utilized to draw an appropriate volume of glue with the action of capillary or suction pressure (if more volume of glue is needed) of about -5 kPa for 20 seconds. Glue is then deposited to the back side of the cantilever (unlike colloidal probe cantilevers) by applying an insufflation pressure of about 5 kPa for 1–2 seconds. The pressure is larger than the friction drag at the interface of glue-micropipette wall. Another micropipette is used to pick up a magnetic microbead by applying a suction pressure to overcome the adhesion at the microbead-substrate interface. The microbead is then released at the target position on the adhesive droplet. Finally, the AFM probe is unloaded and placed in a vacuum oven for 12 hours at 60°C. After all, the magnetic bead is magnetized in a pulse magnetic field (~5 T) along the longitudinal axis of the cantilever. The cantilever is then calibrated for broad modulus range operations in air and liquid.

Preparing modified cantilevers can take some time, which holds true for all type of modified probes, i.e. physically, chemically or biologically. Unlike some of other modified probes, the ones used in this study can be easily commercialized and stored for a relatively long time. When considering the time consumption, however, one should account for the way we are using these probes, to unify the discrete moduli spectra. In other words, with a single probe we cover the moduli range of multiple probes therefore, the time is saved from replacing and recalibrating each probe.

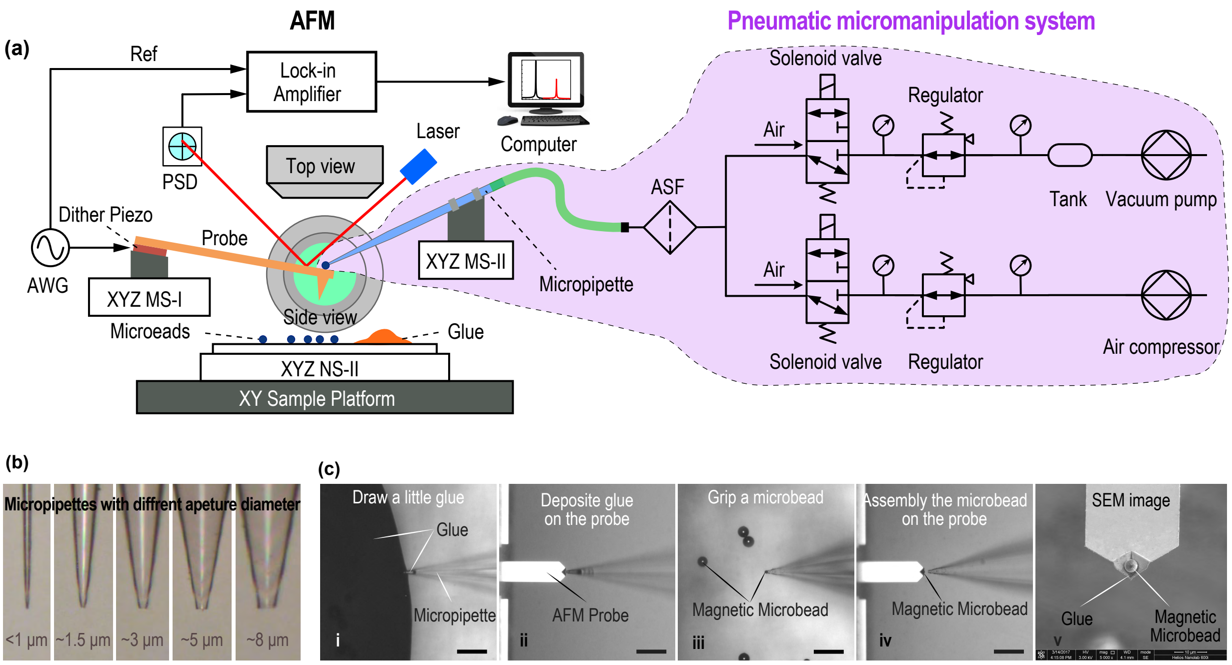

Supplementary Figure 8. Preparation of magnetic microbead probes. (**a**) Schematic diagram of experimental setup includes a pneumatic control system for adhesive bonding of magnetic microbead on the AFM probe. (**b**) Micropipettes with different aperture diameter used to pick-and-place different size of microbeads. (**c**) Optical microscopy images captured during the process of preparation of the magnetic bead probe. Scale bar, 20 μm.

Supplementary References

1. Binnig, G., Quate, C. F. & Gerber, C. Atomic Force Microscope. *Phys. Rev. Lett.* **56**, 930–933 (1986).
2. Martin, Y. & Wickramasinghe, H. K. Magnetic imaging by " force microscopy " with 1000 Å resolution. *Appl. Phys. Lett.* **50**, 1455–1457 (1987).
3. Florin, E. L., Radmacher, M., Fleck, B. & Gaub, H. E. Atomic force microscope with magnetic force modulation. *Rev. Sci. Instrum.* **65**, 639–643 (1994).
4. Phillips, G., Siekman, M., Abelmann, L. & Lodder, J. High resolution magnetic force microscopy using focused ion beam modified tips. *Appl. Phys. Lett.* **81**, 865–867 (2002).
5. Han, W., Lindsay, S. & Jing, T. A magnetically driven oscillating probe microscope for operation in liquids. *Appl. Phys. Lett.* **69**, 4111–4113 (1996).
6. Schemmel, A. & Gaub, H. Single molecule force spectrometer with magnetic force control and inductive detection. *Rev. Sci. Instrum.* **70**, 1313–1317 (1999).
7. Marti, O. et al. Mechanical and thermal effects of laser irradiation on force microscope cantilevers. *Ultramicroscopy* **42**, 345–350 (1992).
8. Kato, N., Suzuki, I., Kikuta, H. & Iwata, K. Force-balancing force sensor with an optical lever. *Rev. Sci. Instrum.* **66**, 5532–5536 (1995).
9. Schäffer, T., Cleveland, J., Ohnesorge, F., Walters, D. & Hansma, P. Studies of vibrating atomic force microscope cantilevers in liquid. *J. Appl. Phys.* **80**, 3622–3627 (1996).
10. Amo, C. A. & Garcia, R. Fundamental high-speed limits in single-molecule, single-cell, and nanoscale force spectroscopies. *ACS Nano* **10**, 7117–7124 (2016).
